# Assessment of the stability of co-amorphous olanzapine in tablets

**DOI:** 10.1080/07853890.2021.1896097

**Published:** 2021-09-28

**Authors:** Nuno F. da Costa, João F. Pinto, Ana I. Fernandes

**Affiliations:** a iMed.ULisboa, Faculdade de Farmácia, Universidade de Lisboa, Lisboa, Portugal; b Centro de Investigação Interdisciplinar Egas Moniz (CiiEM), Egas Moniz Cooperativa de Ensino Superior, Caparica, Portugal

## Abstract

**Introduction:**

Amorphous and co-amorphous (CAM) materials have recently been used to improve oral bioavailability of drugs, by enhancing water solubility and dissolution rate from solid dosage forms [1,2]. However, stress conditions imposed on amorphous systems during production of such dosage forms (e.g. pressure upon tableting), can revert the drug back into the less soluble crystalline form [3]. This work thus presents a methodology to monitor and quantify the fraction of amorphous olanzapine (OLZ) remaining in immediate release OLZ tablets (Table 1).

**Materials and methods:**

Near infra-red (NIR, 9000–4000 cm^−1^) and Fourier-transform infra-red (FTIR, 4000–500 cm^−1^) techniques were used to quantify the fraction of co-amorphous OLZ (30%) with saccharin (SAC; 18%) in formulations containing also calcium phosphate (27%), microcrystalline cellulose (20%) and povidone (5%). Tablets (250 mg) were obtained using a universal testing machine fit with circular punches (7.5 mm Ø), at a constant compression rate of 10 mm/min (*n* = 5); different compression forces (8 and 25 kN) and dwell times (DT; 0 and 20 min) were considered. Evaluation of the impact of the compression conditions on the recrystallization of OLZ from a co-amorphous system was based on a computational model.

**Results:**

Using a 2nd derivative filter to process both NIR and FTIR spectra, the quantification of amorphous olanzapine was possible with a root mean square error of calibration and prediction above 2% (Figure 1). The method was further applied to evaluate the stability of co-amorphous systems after tableting, revealing that no significant recrystallization occurred, i.e. the co-amorphous were stable under the stress conditions applied. **Discussion and conclusions:** The use of NIR and FTIR resulted in high correlation between the predicted values and the expected ones, thus enabling these spectroscopic techniques to be used to monitor the conversion of amorphous into crystalline OLZ forms and thus estimate the impact on drug bioavailability. Understanding of the conversion kinetics will ultimately allow anticipation of the time for complete recrystallization. The model developed has also demonstrated that tablets produced were stable, since no recrystallization was observed.

**Table 1.** Predicted fraction of amorphous OLZ, by NIR and FTIR spectroscopies, in tablets prepared with the co-amorphous system.

ID	NIR	FTIR
CAM physical mixture	100	100
CAM 8 kN 0DT	89	98
CAM 8 kN 20DT	94	94
CAM 25 kN 0DT	95	93
CAM 25 kN 20DT	98	95

**Figure 1.**
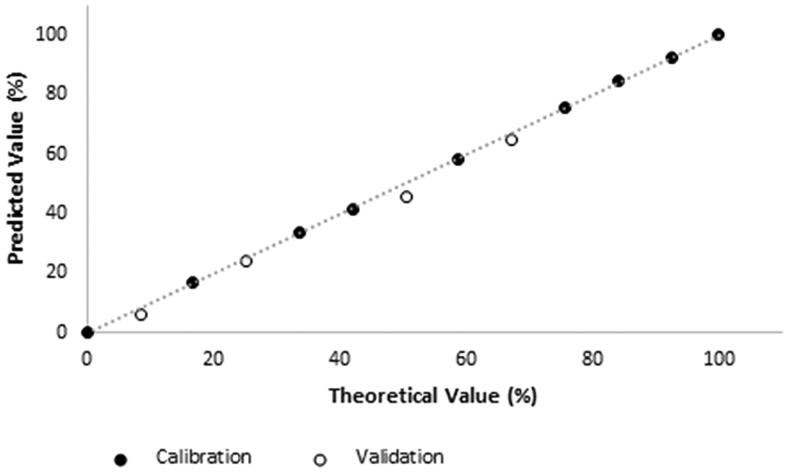
NIR calibration curve used to predict the amorphous fraction of OLZ..
